# Retrospective Cohort Study Demonstrates Tolerance and Adherence to Pea-Based Complete Enteral Formula When Transitioned from a Previous Hypoallergenic Product

**DOI:** 10.3390/nu16193365

**Published:** 2024-10-03

**Authors:** Nicole A. Withrow, Youhanna Al-Tawil, P. J. Patterson, Madden Wilson, Erika Ryan, Vanessa Millovich, Christina J. Valentine

**Affiliations:** 1 Kate Farms, Inc., Goleta, CA 93111, USA; yaltawil@giforkids.com (Y.A.-T.); vanessa.millovich@katefarms.com (V.M.); valentine2@arizona.edu (C.J.V.); 2 GI for Kids, Knoxville, TN 37922, USA; pjpatterson@giforkids.com (P.J.P.); mwilson@giforkids.com (M.W.); 3 Baxter International, Deerfield, IL 60015, USA; erika.breitfeller@gmail.com; 4 Department of Pediatrics and Nutrition, The University of Arizona, Tucson, AZ 85721, USA

**Keywords:** cow’s milk protein allergy, plant based, enteral nutrition, extensively hydrolyzed formula, yellow pea protein

## Abstract

Background: Plant-based formulas have become increasingly popular due to their health benefits, environmental concerns, cultural beliefs, improved palatability, and decreased cost. A retrospective chart review of medically stable children transitioning from a hypoallergenic formula to a yellow pea protein plant-based formula (PPPBF) was included. This study aimed to assess gastrointestinal tolerance, weight changes, and adherence to receiving a unique PPPBF. Methods: Healthcare providers (HCPs) from pediatric clinics across the United States who requested increased PPPBF samples between the dates of 1 November 2021and 31 January 2022 and again from 1 February 2022 to 15 April 2022 inputted survey data. The HCPs selected participants based on the inclusion criteria. Results: Seventy-three completed patient surveys were included of children (ages 1–18 years old, 41% females, 59% males). After the transition to PPPBF, 38.4% experienced improvement in GI tolerance, 56.2% experienced no change, and 5.5% reported worsening GI tolerance. There was a 95% adherence rate, and 98.6% reported no adverse reactions or allergic manifestations after formula transition. Conclusions: Transitioning from a hypoallergenic formula to a PPPBF showed a trend toward stable GI tolerance, weight gain or stability, and adherence. A PPPBF offers a first-choice option for children who are on hypoallergenic formulas due to intolerance.

## 1. Introduction

Young children who are diagnosed with a cow’s milk protein allergy (CMPA) generally require a hypoallergenic, extensively hydrolyzed formula [1]. The most common food allergy in infants and young children is CMPA, with a prevalence ranging from 0.54 to 7.5% depending on geographic region, age of the individual, and diagnostic criteria [2]. Cow’s milk protein allergies are classified as IgE-mediated or non-IgE-mediated. An IgE-mediated CMPA is typically easier to diagnose since symptoms appear quickly after consumption, while a non-IgE-mediated CMPA is more challenging to diagnose because of a delay in symptoms [3]. It is not uncommon for children to be allergic to more than one cow’s milk allergen, such as whey and casein [4]. Symptoms of CMPA include diarrhea, vomiting, nausea, rashes, breathing problems, and anaphylaxis and can vary in severity [5]. Fortunately, many children outgrow the allergy, but the reasons remain unclear. There is general agreement within the research literature that the standard of care for a CMPA is total avoidance of cow’s milk protein; therefore, hypoallergenic, extensively hydrolyzed formulas (ETFs) are recommended [2,5,6].

**Figure 1:**
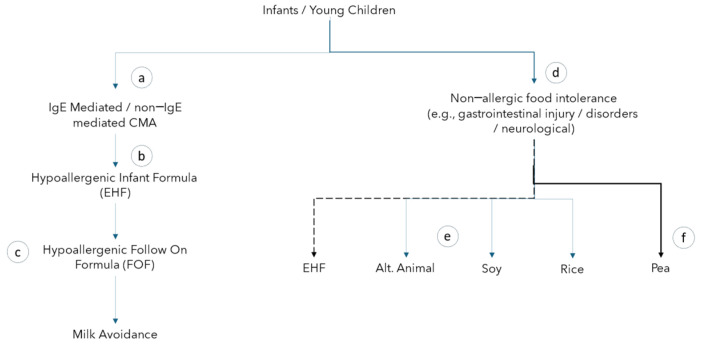
Feeding options for young children with and without diagnosed cow milk protein allergy.

Research suggests that 75% of children outgrow their CMPA by age five, but those still allergic require long-term management by avoiding cow’s milk proteins in food and beverages [7]. Current clinical practice steers practitioners to prescribe a hypoallergenic formula even when a child is not diagnosed with a CMPA (Figure 1). Figure 1 illustrates common clinical practices for prescribing formulas to infants and children, and it was created using the author’s experience

Children with gastrointestinal disorders, intestinal injury, or other complex medical conditions such as neurological conditions are often prescribed a hypoallergenic formula despite a paucity of evidence to support that this is necessary for children without a diagnosed CMPA. Not only are these formulas reported to be less palatable, but they are also more expensive when compared to alternative formulas [8]. Different formulas that target the etiology behind the intolerance, such as protein source, osmolality, sweeteners, and lack of fiber, could be a viable option to improve tolerance and be more palatable and cost-effective.

Several alternative formulas are available to treat children who need a specialty formula. Alternative formulas from other animal milk or plant-based proteins have emerged out of necessity and consumer interest. Interest in non-cow milk formula has increased due to its potential utility [9]. Research is needed to understand if alternative animal milk formulas such as goat, sheep, camel, donkey, and horse can treat and prevent CMPA and food intolerances [10]. Plant-based protein formulas have become increasingly popular due to their health benefits, environmental and climate concerns, cultural beliefs, improved palatability, and decreased cost. Soy protein isolate was first used in infant formulas over 60 years ago for infants with severe CMPA or colic [11]. Soy allergy is less common, affecting approximately 0.3–0.4% of young children, but it has been suggested that 10% of CMPA patients develop a soy protein allergy [12,13]. There have been some concerns about nutritional deficiencies and the phytate and phytoestrogen content in soy, but today, soy-based formulas are considered a safe alternative to cow’s milk formulas [10]. Rice-based formulas have become a plant-based option because they do not contain animal proteins and have been tolerated in infants allergic to cow’s milk and soy [14]. According to the World Allergy Organization (WAO), a hydrolyzed rice formula is a viable option for non-IgE-mediated CMPA and other food intolerances [2]. Hydrolyzed rice formulas are well tolerated in most children and support growth and development [14]. Rice-based formulas have become more popular because they are less expensive than other formulas [1]. Yellow pea protein (*Pisum sativum*) formulas have emerged as a potential alternative for those with CMPA, other allergic diseases, GIDs, enteropathies, and motility disorders. Yellow pea protein is not included in the list of the top nine common food allergens recognized by the Food and Drug Administration (FDA). These major food allergens include milk, eggs, fish, crustacean shellfish, tree nuts, peanuts, wheat, soybeans, and, most recently, sesame seeds [15]. The yellow pea has been reported to have a role in disease prevention, postprandial glycemic response, and weight and blood pressure control, and it has anabolic properties [16,17]. It has been postulated that these benefits are due to yellow peas’ nutritional and non-nutritional components [18]. It is a good source of protein, complex carbohydrates, vitamins, and minerals and has naturally occurring phytochemicals and phytonutrients [19]. The yellow pea is readily available and nutrient-dense. 

The commercially available yellow PPPBF used in this study is organic, non-GMO project verified, near isotonic osmolality, contains no artificial or non-nutritive sweeteners, and has organic agave inulin. The yellow pea protein in the PPPBF is cleaned and processed without using chemical solvents. This process preserves the peptide structure, so the flavor profile is not changed, and a purified pea protein isolate is the result [20]. The yellow pea protein isolate used in this formula is a high-quality vegetable protein. It contains the nine essential amino acids with added L-cysteine in the correct proportions to meet the 2–5-year-old essential amino acid requirement [17]. It provides a protein digestibility-corrected amino acid score (PDCAAS) of 1.0. In addition, the yellow pea protein has natural emulsification properties, which contributes to a less bitter taste than other plant-based proteins [21]. There is a naturally occurring source of arginine, which is essential for cell growth, proliferation, and wound healing, and it becomes a conditionally essential amino acid during acute stress and illness [22]. No clinical studies have examined the GI tolerance and efficacy of transitioning patients from a hypoallergenic formula to a yellow PPPBF.

The primary aim of this study was to assess gastrointestinal (GI) tolerance, weight changes, and adherence after transitioning from a hypoallergenic formula to a yellow PPPBF. 

## 2. Materials and Methods

### 2.1. Study Design

This was a retrospective review of electronic medical records approved by Advarra, Columbia, MD 21044, USA, a centralized institutional review board (IRB) under exempt status to assess growth and tolerance in pediatric patients who transitioned from a hypoallergenic formula to a yellow PPPBF.

### 2.2. Participants

Study invitations were emailed to healthcare providers in pediatric outpatient care centers across the United States who requested increased PPPBF samples between the dates of 1 November 2021 and 31 January 2022 and again from 1 February 2022 to 15 April 2022. The healthcare providers selected participants based on the inclusion criteria. Inclusion criteria consisted of participants 1 through 18 years of age who transitioned from a hypoallergenic formula to a yellow PPPBF within 12 months of the recruitment start date, had an initial assessment or progress note 2–4 weeks before the transition, or on the day the transition was recommended, and post formula transition. All participants were medically stable with no acute illness or risk of hospitalization and consumed the formula by mouth or were tube-fed for supplemental or sole-source nutrition.

### 2.3. Procedures

Healthcare providers retrospectively reviewed electronic medical records (EMRs) and collected information in the HIPPA-compliant Research Electronic Data Capture (REDcap, Vanderbilt University, Nashville TN 37235, USA) system. The HCPs were instructed to complete the survey questions as written to ensure consistent reporting across healthcare providers. The survey link was available for two weeks, allowing the healthcare provider to enter patient information at their convenience (12 May 2022–26 May 2022). Healthcare providers entered their patients’ de-identified demographic information, clinical characteristics, growth (weight changes), tolerance, adherence, and adverse reactions to the unique yellow PPPBF (Kate Farms^TM^, Goleta CA 93111, USA) following the transition. Any GI intolerances were reported as abdominal pain, reflux, diarrhea, constipation, bloating, nausea, vomiting, and early satiety. For this study, GI tolerances were reported as changes in symptoms. For example, one question within the survey asked, “When using extensively hydrolyzed/amino acid-based formula, did the patient experience GI intolerance?” (Please select all that apply) (choices = the patient did not have any GI intolerance to hypoallergenic formula; early satiety; vomiting; nausea; bloating; constipation; diarrhea; reflux; abdominal pain). Two additional questions on GI tolerance included “Did the transition from extensively hydrolyzed/amino acid-based formula to Kate Farms formula result in improved GI tolerance and decrease in GI symptoms such as abdominal pain, reflux, diarrhea, constipation, bloating, nausea, vomiting, early satiety?” and if the HCP selected either “Yes—GI tolerance improved” or “No—GI tolerance worsened”, they were asked subsequent questions on which of the following GI symptoms improved and which of the following GI symptoms worsened.

### 2.4. Statistical Analyses

Descriptive statistics were calculated for the clinicians’ and patients’ demographic and clinical characteristics. Clinician demographic information included the clinician’s role and medical specialty, and patient demographic characteristics included sex, age, and primary medical diagnosis. Clinical characteristics included the primary reason for the hypoallergenic formula, the type of hypoallergenic formula, the primary reason for transitioning to the yellow PPPBF, the method of feeding, and the length of time on the PPPBF (>4 weeks). Weight changes before and after the transition were recorded as raw data, and adherence was measured by receiving ≥ 75% of the prescribed PPPBF regimen. Frequencies and percentages were calculated for GI tolerance, growth outcomes, adherence, and adverse reactions. Adverse reactions were recorded. A sample size and power calculation were not performed due to being a sample of convenience.

## 3. Results

Of the 1312 clinicians who were emailed the electronic survey, 79 surveys were completed, but six were excluded from the final data analysis due to the patient’s acute illness or hospitalization during the formula transition period. Table 1 shows the demographic characteristics of the clinicians who participated. Of the 73 clinicians who were included in the final data analysis, 52 (71.2%) were registered dietitians, 2 (2.7%) were nurses, 17 (23.3%) were research coordinators, and 2 (2.7%) were nurse practitioners or medical doctors. The clinicians were in seventeen different states, with the majority specializing in gastroenterology (61.6%) (Table 1). Of the 73 completed patient surveys, the sample consisted of 59% males and 41% females, with a mean age of 4.7 years (1–18 years old).

Medical diagnoses were included as a demographic variable but did not impact the inclusion. The top five primary medical diagnoses included developmental delays (13.7%), failure to thrive (12.3%), dysphagia (12.3%), cow’s milk protein allergy (8.2%), and gastroparesis (8.2%) (Table 2). Before transitioning to PPPBF, 8% of the patients consumed extensively hydrolyzed formula, and 92% consumed an amino acid-based formula. Only 5 of the 73 patients (6.8%) received a fiber-containing hypoallergenic formula at baseline. The remaining 93.2% of the patients received fiber-free formula prior to transitioning to a fiber-containing PPPBF. Patients were consuming a hypoallergenic formula prior to transition due to GI intolerance to their previous formula (31.5%), cow’s milk protein allergy (28.8%), dysmotility (16.4%), malabsorption of their previous formula (13.7%), other allergies (2.7%), and 6.8% for other reasons.

Interestingly, clinicians reported that 46.6% of patients still experienced GI intolerances while consuming a hypoallergenic formula at baseline (Table 3). The majority (57.5%) of patients transitioned to the PPPBF because their formula was no longer available due to the national formula shortage, while 19% transitioned due to poor weight gain, 11% due to intolerance, 8% transitioned from infant formula at 12 months of age, and 4% was due to the parental preference for ingredients in the yellow PPPBF. Overall, 34 patients (46.6%) transitioned to Kate Farms^TM^ Pediatric Peptide 1.0, 14 (19.2%) transitioned to Kate Farms^TM^ Pediatric Peptide 1.5, and 25 (34.2%) transitioned to Kate Farms^TM^ Pediatric Standard 1.2. In total, 59%of the patients received tube feeds, whereas 27.4% were on oral feeds and 13.7% received both tube and oral feeds.

After transitioning to the PPPBF, 38.4% experienced improvement in GI tolerance, 56.2% experienced no change in GI tolerance, and 5.5% reported that GI tolerance had worsened. Out of the four (5.5%) patients with worsened GI tolerance after formula transition, diarrhea and bloating were the two reported symptoms (Table 4). Weight changes were analyzed: 64.4% gained weight, 30% had weight stability, and 5.5% experienced weight loss after formula transition (Table 4). There was a 95% adherence rate in all patients who transitioned to PPPBF, defined as receiving greater than or equal to 75% of the prescribed nutrition regimen (Table 4). One patient was reported to experience an adverse reaction after transitioning to the PPPBF, which was reported as having increased diarrhea. A total of 98.6% of patients experienced no adverse reactions or allergic manifestations after formula transition (Table 4).

Observations were made in a sub-cohort of patients who were previously diagnosed with allergic disease; 23 (31.5%) of the patients had CMPA or other allergies as the primary reason for being on a hypoallergenic formula at baseline. In this sub-cohort of patients, it was noted that 56.5% reported GI intolerance when using a hypoallergenic formula at baseline. Of the 23 patients, 100% tolerated the transition to PPPBF, 48% experienced improvement in GI tolerance after the transition, and 52.2% experienced no change in GI tolerance. In addition, 61% of patients gained weight, 30% maintained weight, and 8.7% lost weight after the transition. Adherence was consistent with the findings from the total cohort at 96% after the transition. Lastly, no adverse reactions or allergic manifestations were noted after the transition from a hypoallergenic formula to PPPBF in this allergic disease subset of the total cohort of patients.

## 4. Discussion

This retrospective cohort examination is the first to our knowledge to examine the tolerance, weight changes, and adherence of transitioning pediatric patients with various medical conditions from a hypoallergenic formula to a yellow PPPBF. The participants demonstrated positive trends with GI tolerance, weight gain or stabilization, and adherence to the yellow PPPBF transition.

The tolerance etiology could be the novel attributes of the yellow pea as compared to the other dairy products, including source/peptide size, osmolality, fiber, and non-nutritive sweetener content. The PPPBF provides a source of protein from intact or partially hydrolyzed yellow pea protein with naturally occurring arginine. Hypoallergenic formulas are commonly formulated without fiber, even though constipation and diarrhea are often reported in enterally fed patients [23]. Only 6.8% of patients received fiber in their hypoallergenic formula before transitioning to the PPPBF, which provided a moderate amount of fiber. The PPPBF contains organic agave inulin, a fermentable soluble fiber. Agave inulin has a low glycemic index, and it has been shown to have a role in maintaining a healthy microbiome [24,25,26]. There is emerging literature surrounding the consequences of dietary fiber deprivation [27,28]. Dietary fiber provides food to the microbiota in the gut, which allows the gut microbiota to ferment the fiber into short-chain fatty acids (SCFAs) [27,28]. The intestinal mucosal layer protects the gut and comprises 80% glycans, which feed the gut microbiota when fermentable fiber is unavailable [29]. This utilization of mucus glycoproteins as ‘food’ due to dietary fiber deprivation leads to increased epithelial access and mucosal degradation. Lastly, non-nutritive sweeteners or artificial sweeteners are present in many hypoallergenic pediatric formulas. There is emerging literature on the effects of sweeteners on the gut microbiota and GI tolerance. In a 2019 review of experimental studies and clinical trials, it was reported that saccharin, sucralose, and stevia change the composition of the gut microbiota; sucralose consumption was associated with an under-representation of beneficial bacteria [30]. The authors concluded that clinicians should exercise caution regarding non-nutritive sweetener consumption. Non-nutritive sweetener intake has also been demonstrated to modify the expression of hormones, which consequently impact gastrointestinal motility. In addition to the impact on gastric motility, functional GI disorder symptoms are linked to non-nutritive sweetener intake [31]. One study showed a significant increase in functional GI disorder symptoms in patients consuming non-nutritive sweeteners daily as a dietary intervention [32].

Due to the nature of a retrospective chart review, generalizing the results to a larger population should be cautioned. The results of this study showed a positive trend toward stable GI tolerance, weight gain or stability, and adherence in the studied cohort. Another limitation of this study was that the PPPBF used has not been classified as hypoallergenic since it has not been formally studied in a double-blinded, placebo-controlled trial; however, many patients are often on hypoallergenic formulations without a diagnosis of CMPA. This source could potentially enable children to tolerate an enteral product without having to incur so many switches and demonstrates great adherence and satisfaction. Other limitations of this study include a small sample size and selection bias of the HCPs. However, selection bias was addressed by having detailed inclusion criteria. The small sample size may have been due to the increased mental stress experienced by these HCPs as they were on the front lines of the patient, caregiver, and insurance complaints and issues related to the elemental formula shortage. Additionally, HCPs were recruited to participate via email at the beginning of the two-week study period and were not contacted in any other way.

There were several strengths in this study. Incomplete data did not exist due to rules within REDcap, which did not allow the HCPs to continue entering information if certain criteria for that patient did not exist; additionally, all questions required an answer to continue the survey and to submit it. Recall bias was minimized by including HCPs who routinely followed the participants, and the recorded data came directly from an electronic medical record instead of relying on the participant’s recall. 

## 5. Conclusions

This retrospective chart review demonstrated PPPBF as a viable transition formula for this cohort of pediatric patients who previously received a hypoallergenic formula. Transitioning from a hypoallergenic formula to a PPPBF demonstrated a trend towards stable GI tolerance, weight gain or stability, and adherence. It offers a first-choice option for children on hypoallergenic formulas due to intolerance. Future research is needed to understand the safety and efficacy in children with a diagnosed cow’s milk protein allergy.

## Figures and Tables

**Table 1:** Clinician demographic characteristics.

Variable	n	%
**Clinician role:**		
Registered dietitian	52	71
Nurse	2	3
NP/MD	2	3
Research coordinator	17	23
**Medical specialty:**		
Gastroenterology	45	62
General pediatrics	19	26
Cystic fibrosis	1	1
Neonatology	1	1
Other	7	1

**Table 2:** Demographic and clinical characteristics of study sample.

Variable	n	%
**Patient:**		
Female	30	41
Male	43	59
**Age (yrs):**		
1–6	57	78
7–12	10	14
13–18	6	8
**Primary medical diagnosis:**		
Gastroparesis	6	8
FTT	9	12
Malnutrition	3	4
Cystic fibrosis	1	1
Cerebral palsy	6	8
Developmental delay	10	14
Dysphagia	9	12
Short bowel syndrome	4	6
Congenital heart disease	4	6
Multiple food allergies	2	3
Cow’s milk protein allergy	6	8
Eosinophilic esophagitis (EoE)	1	1
Liver disease	2	3
Other	10	14

**Table 3:** Primary reasons for EN and the types of formula consumed by patients (n = 73).

Variable	n	%
**Reason for hypoallergenic formula:**		
Malabsorption	11	15
Dysmotility	13	18
GI intolerance to previous formula	25	34
CMPA	21	29
Other allergies	2	3
Missing	1	
**Patient route of administration:**		
Oral	20	27
Tube feeding	43	59
Oral and tube feeding	10	14
**Patient baseline formula:**		
Extensively hydrolyzed infant	6	8
Amino acid-based infant	19	26
Amino acid-based infant with fiber	1	1
Amino acid-based junior	43	59
Amino acid-based junior with fiber	4	6
**Patient transition PPPBF:**		
Kate Farms Pediatric Standard 1.2	25	34
Kate Farms Pediatric Peptide 1.0	34	47
Kate Farms Pediatric Peptide 1.5	14	19
**Patient GI intolerance at baseline:**		
Abdominal pain	5	7
Reflux	14	19
Diarrhea	4	6
Constipation	20	27
Bloating	7	10
Nausea	1	1
Vomiting	7	10
Early satiety	4	6
No GI intolerance	39	53

**Table 4:** Patient outcomes after transition from a hypoallergenic formula to a pea protein plant-based formula (PPPBF) (n = 73).

Variable	n	%
**GI Symptoms:**		
Improved	28	38
No change	41	56
Worsened	4	6
**Weight changes:**		
Gain	47	64
No change	22	30
Loss	4	6
**Adherence:** *		
Yes	69	95
No	4	6
**Adverse reaction:** **		
None	72	99
Other (diarrhea)	1	1

* Adherence = >75% intake. ** Adverse reaction defined = skin manifestations, allergic reaction, and/or blood in stool.

## Data Availability

Data can be requested by contacting corresponding author, Nicole A. Withrow (nikki.withrow@katefarms.com).
